# Pediatric Primary Care Provider Comfort with Mental Health Practices: A Needs Assessment of Regions with Shortages of Treatment Access

**DOI:** 10.1007/s40596-021-01434-x

**Published:** 2021-03-30

**Authors:** Amie F. Bettencourt, Rebecca A. Ferro, Jami-Lin L. Williams, Kainat N. Khan, Rheanna E. Platt, Sarah Sweeney, Kelly Coble

**Affiliations:** 1 Johns Hopkins School of Medicine, Baltimore, MD USA; 2 University of Maryland School of Medicine, Baltimore, MD USA

**Keywords:** Primary care providers, Child psychiatry, Provider comfort, Pediatrics, Integrated care

## Abstract

**Objectives:**

Nearly 50% of children with a mental health concern do not receive treatment. Child Psychiatry Access Programs like Behavioral Health Integration in Pediatric Primary Care (BHIPP) address regional shortages of mental health treatment access by providing training and consultation to primary care providers (PCPs) in managing mental health concerns. This study assessed PCPs’ comfort with mental health practices to inform expansion of BHIPP services.

**Methods:**

Pediatric PCPs in 114 practices in three rural regions of Maryland were recruited to participate in a survey about their comfort with mental health practices and access to mental health providers for referral. Descriptives, Friedman’s test, and post hoc pairwise comparisons were used to examine survey responses.

**Results:**

Participants were 107 PCPs. Most respondents were physicians (53.3%) or nurse practitioners/physician’s assistants (39.3%). Friedman’s test, *χ*^2^(7)= 210.15, *p*<.001, revealed significant within and between-group differences in PCP comfort with mental health practices. Post hoc pairwise comparisons indicated greater comfort providing mental health screening and referrals compared to prescribing psychiatric medications, providing psychoeducation or in-office mental health interventions. A Wilcoxon-signed rank test showed significantly more respondents agreed they could find a therapist than a psychiatrist in a timely manner, *Z*= −5.93, *p*<.001.

**Conclusions:**

Pediatric PCPs were more comfortable with providing mental health assessment and referrals than treatment. However, PCPs reported difficulty finding therapists and psychiatrists for their patients. Findings underscore the need for longitudinal training to increase PCP comfort with mental health treatment. Additionally, strategies such as telepsychiatry are needed to address the disproportionate need for child psychiatrists.

Up to 20% of children in the USA experience a mental health concern in a given year, and this rate has steadily increased over time [1]. However, there is a significant gap between the need for and availability of pediatric behavioral health services, particularly in regions outside of major metropolitan areas [2]. As a result, pediatric primary care providers (PCPs) are responsible for a substantial volume of mental health care [3]. A growing number of regions and states have developed child psychiatry access programs to support PCPs in meeting the mental health needs of their patients. These programs are now operating in >30 states in the USA [4].

While the services provided by child psychiatry access programs vary based on differing policy, financial, and workforce contexts, programs typically include training, case-based consultation, and resource/referral assistance [5]. Training opportunities range in intensity from standalone hour-long programs to intensive longitudinal programs [4, 6]. Several programs have documented improved PCP comfort with identifying and managing mental health concerns, [7–9] particularly when PCPs engage in both training and case-based consultation [10]. Others have observed within-state variation in program utilization by region (e.g., providers in upstate New York more likely to utilize child psychiatry access program services compared to other New York regions), practice and provider characteristics (e.g., panel size, distance to child psychiatry access program “hub,” motivation to participate in additional training) [6, 10, 11]. Given variability in program utilization and provider availability within statewide jurisdictions, it is critical to understand the specific needs of regions (e.g., rural vs urban) or subgroups (e.g., large vs small practices), particularly in the case of rural/remote regions with the most pronounced shortages of mental health providers.

Behavioral Health Integration in Pediatric Primary Care (BHIPP) is a statewide child psychiatry access program that provides training, telephone consultation, and referral assistance to pediatric PCPs to strengthen their capacity (knowledge, comfort, skills) to address the mental health needs of their patients. In 2019, BHIPP was funded by the Health Resources and Services Administration to increase the availability and accessibility of pediatric behavioral health treatment through the addition of tele-mental health and web-based longitudinal training services. The initial focus of the project was on remote/rural regions of Maryland with the greatest shortages of mental health providers, particularly child and adolescent psychiatrists (CAPs): Southern Maryland, Western Maryland, and Lower Eastern Shore [12]. The purpose of this study was to conduct a needs assessment to understand pediatric PCPs’ comfort with managing mental health problems in their practices and access to local mental health providers to inform BHIPP’s service expansion.

## Methods

PCPs at all known practices (*N* = 114) in three regions (Southern Maryland, Western Maryland, and Lower Eastern Shore) that provide pediatric primary care services, including PCPs with and without a history of engagement with BHIPP, were recruited to participate in a survey about their capacity to address their patients’ mental health concerns. According to the census, the target regions all have low population densities (Southern Maryland: 344 people per square mile, Western Maryland: 182, Lower Eastern Shore: 129), and two of the three have median household incomes below the national average (National: $61,937, Southern Maryland: $96,283, Western Maryland: $51,746, Lower Eastern Shore: $51,090) and greater proportions of people living in poverty than the national average (National: 11.8%, Southern Maryland: 7.2%, Western Maryland: 14.3%, Lower Eastern Shore: 17.2%). Additionally, although Southern Maryland is within close proximity to a major metropolitan area, Western Maryland and Lower Eastern Shore are more geographically isolated. These regions were targeted for a planned expansion of BHIPP tele-mental health services in light of these characteristics and because of a significant shortage of mental health providers in each region, with an average of 3.6 psychiatrists/10,000 children across regions [12] (Southern Maryland:1.2, Western Maryland: 4.6, Lower Eastern Shore: 4.7).

Study procedures were approved by the University’s IRB. PCPs were recruited through a combination of mail, telephone calls, fax, email, and in-person visits to practices. Written or oral consent was collected during survey completion. All completed surveys were reviewed and, if completed on paper, entered into Qualtrics.

The survey, developed by the authors, comprises three sections. Section one asks for general information about the PCP and their respective practice(s), including contact and demographic information (i.e., race, gender), provider type (e.g., physician, nurse practitioner) and specialty, practice characteristics (e.g., patient volume, percentage of pediatric patients), whether onsite mental health services are available and if so, who (e.g., psychologist, social worker) provides those services. Section two includes an eight-item scale (see Table 2 for items) measuring providers’ perceived comfort with evaluation and treatment of pediatric mental health concerns on a scale from 1 (*very uncomfortable*) to 4 (*very comfortable*). This scale has strong internal consistency (*α* = .89). Section three includes two items (Table 2) assessing PCPs’ perceived ability to find mental health providers in a timely manner on a scale from 1 (*strongly disagree*) to 4 (*strongly agree*).

Descriptives were used to examine survey responses. A Friedman’s test was conducted to determine whether there were significant differences among PCP comfort items. Post-hoc pairwise comparisons were conducted using the Wilcoxon-signed rank test to determine which comparisons were significant. Since multiple comparisons (25 post-hoc comparisons) were completed, a Bonferroni correction was made (.05/25) and the adjusted significance level of *p<*.002 was used. A separate Wilcoxon-signed rank test was completed to examine differences between providers’ ease of finding therapists versus CAPs.

## Results

Surveys were completed by 107 pediatric PCPs from 49% of eligible practices in target regions. The sample was majority female (75.7%) and White (63.6%). Respondents had been in practice for an average of 15.0 years and the majority reported a weekly caseload of more than 50 patients. Most respondents were physicians (53.3%) or nurse practitioners/physician’s assistants (39.3%), and the majority specialized in Pediatrics (51.0%) or Family Practice (35.5%). However, provider type, *χ*^2^ (8)=22.20, *p*=.005, and specialty *χ*^2^ (10)=25.24, *p*=.005, differed significantly by region, with respondents from Southern Maryland including more nurses and those from a specialty other than Pediatrics or Family Practice, and respondents from Western Maryland including more nurse practitioners/physician’s assistants and providers specializing in Family Practice. See Table 1 for provider characteristics.

**Table 1:** Provider characteristics by region

Provider Characteristics	Overall *N* (%)	Southern Maryland *N* (%)	Western Maryland *N* (%)	Lower Eastern Shore *N* (%)	Chi-Square value
All providers	107 (100%)	41 (38.3%)	44 (41.1%)	22 (20.6%)	
Average years in practice (M/SD)* (*N*=97)	15.0 (11.7)	14.7 (10.2)	12.7 (12.0)	19.4 (13.0)	
Average years at current site (M/SD) (*N*=92)	8.6 (9.2)	7.2 (7.4)	9.3 (11.5)	9.7 (6.8)	
Provider type					18.86**
Physician (MD/DO degrees)	67 (53.3%)	23 (56.1%)	20 (45.5%)	14 (63.6%)	
Nurse practitioner/physician’s assistant	42 (39.3%)	10 (24.4%)	24 (54.5%)	8 (36.4%)	
Nurse	8 (7.5%)	8 (19.5%)	-	-	
Provider specialty					14.76**
Pediatrics	55 (51.4%)	22 (53.7%)	18 (40.9%)	15 (68.2%)	
Family practice	38 (35.5%)	9 (22.0%)	23 (52.3%)	6 (27.3%)	
Other	14 (13.1%)	10 (24.4%)	3 (6.8%)	1 (4.5%)	
Provider weekly caseload					11.66
≤ 50	17 (16%)	6 (14.6%)	10 (22.7%)	1 (4.5%)	
51–75	29 (27.4%)	15 (36.6%)	9 (20.5%)	5 (22.7%)	
76–100	45 (42.5%)	12 (29.3%)	20 (45.5%)	13 (59.1%)	
101+	15 (14.2%)	8 (19.5%)	4 (9.1%)	3 (13.6%)	
Missing	1 (0.9%)	-	1 (2.3%)	-	
Provider gender					6.88
Female	81 (75.7%)	36 (87.8%)	31 (70.5%)	14 (63.6%)	
Male	25 (23.4%)	5 (12.2%)	12 (27.3%)	8 (36.4%)	
Missing	1 (0.9%)	-	1 (2.3%)	-	
Provider race/ethnicity					9.84
White	68 (63.6%)	24 (58.5%)	29 (65.9%)	15 (68.2%)	
Asian	13 (12.1%)	7 (17.1%)	3 (6.8%)	3 (13.6%)	
African American	11 (10.3%)	5 (12.2%)	5 (11.4%)	1 (4.5%)	
Other	6 (5.6%)	2 (4.9%)	1 (2.3%)	3 (13.6%)	
Missing	9 (8.4%)	3 (7.3%)	6 (13.6%)	-	

Note: Percentages included in the all-providers row are based on the total sample whereas percentages in all subsequent rows are based on the specific region

*M/SD = mean/standard deviation

**p <.01

Table 2 displays frequencies for provider comfort and mental health treatment access items. Friedman’s test revealed significant within-group differences across all regions in providers’ comfort with specific mental health practices, Friedman’s *χ*^2^ (7)=210.15, *p*<.001. Post-hoc pairwise comparisons indicated that providers reported greater comfort providing mental health referrals (Zs= −6.50, −5.60, −6.45, −5.93), asking families about mental health problems (Zs= −6.89, −6.25, −6.71, −6.49), asking about major life changes/stressors (Zs= −6.58, −5.77, −6.42, −6.32), and using screening tools (Zs= −6.57, −5.22, −6.04, −5.69) compared to prescribing psychiatric medications (*p*<.001), providing psychoeducation (*p*<.001), providing in-office mental health interventions (*p*<.001), and supporting other PCPs in addressing mental health problems (*p*<.001). Additionally, providers were more comfortable providing psychoeducation (*Z*= −4.27, *p*<.001) than prescribing psychiatric medication. When considered by region, there were between-group differences in comfort with prescribing psychiatric medication, *χ*^2^ (4)=9.78, *p*=.044, such that more providers in Southern Maryland (33.3%) reported being very uncomfortable prescribing psychiatric medication compared to Western Maryland (7.1%) or Lower Eastern Shore (4.5%). No other regional differences were found.

**Table 2:** Provider comfort with specific mental health practices and treatment access (*N*=107)

Primary care provider comfort questions	Very uncomfortable *N* (%)	Somewhat comfortable/uncomfortable *N* (%)		Very comfortable *N* (%)
Providing referrals to mental health providers (*N*=103)	8 (7.8%)	26 (25.2%)		69 (67.0%)
Asking families about major life changes or stressors (*N*=104)	3 (2.9%)	36 (34.6%)		65 (62.5%)
Asking families about mental health problems (*N*=104)	3 (2.9%)	38 (36.5%)		63 (60.6%)
Using screening tools to identify mental health problems (*N*=102)	6 (5.9%)	38 (37.3%)		58 (56.9%)
Educating families about the nature, course, and treatment of mental health problems (*N*=103)	4 (3.9%)	74 (71.8%)		25 (24.3%)
Providing support to other PCPs in addressing mental health problems (*N*=101)	14 (13.9%)	65 (64.4%)		22 (21.8%)
Prescribing psychiatric medications (*N*=102)	19 (18.6%)	66 (64.7%)		17 (16.7%)
Providing in-office mental health interventions (*N*=103)	6 (5.8%)	80 (77.7%)		17 (16.5%)
Access to mental health provider questions	Strongly disagree *N* (%)	Disagree *N* (%)	Agree *N* (%)	Strongly agree N (%)
I can find a therapist/counselor in a timely manner (*N*=104)	30 (28.0%)	38 (35.5%)	27 (25.2%)	9 (8.4%)
I can find a psychiatrist in a timely manner (*N*=104)	49 (45.8%)	39 (36.4%)	13 (12.1%)	3 (2.8%)

Providers also responded to questions about the availability of mental health services (see Table 2). A Wilcoxon-signed rank test indicated that significantly more respondents agreed/strongly agreed they could find a therapist compared to a CAP in a timely manner, *Z*= −5.93, *p*<.001. Among respondents, 33.6% agreed/strongly agreed that they could find a therapist/counselor whereas only 14.9% agreed/strongly agreed that they could find a CAP. There were significant regional differences in access to therapists, *χ*^2^ (8)=28.92, *p*<.001, such that more providers in Southern Maryland (90.3%) disagreed or strongly disagreed that they could find a therapist in a timely manner compared to the Lower Eastern Shore (54.6%) and Western Maryland (43.1%). There were also significant regional differences in access to CAPs, *χ*^2^ (8)=27.25, *p*=.001, such that more providers in Southern Maryland (95.2%) and Western Maryland (77.3%) disagreed or strongly disagreed that they could find a CAP in a timely manner compared to the Lower Eastern Shore (68.2%). Across all regions, 37.4% of providers reported having some mental health services at their practice. Of those with mental health services on-site, providers reported having a psychologist (45.0%), counselor (42.5%), social worker/case manager (27.5%), PCP who provides mental health services (25.0%), telepsychiatrist (12.5%), or psychiatrist (12.5%).

## Discussion

Across the three regions, PCPs surveyed were more comfortable providing mental health assessment and referral than psychoeducation or direct interventions. However, PCPs reported difficulty finding therapists and CAPs for their patients. These findings are in line with prior work, which has found that PCPs view their role in mental health care as limited to screening and diagnosis, with the exception of ADHD treatment—about which providers still report discomfort initiating medication, particularly with more severe cases or when comorbidities are present [13, 14]. However, consistent with PCP perceptions in this study, there remains a significant shortage and maldistribution of behavioral health providers, particularly CAPs, and this shortage is more pronounced in rural/remote communities, like those surveyed in this study, where delays in access to care may result in poor patient outcomes [15]. This lack of access necessitates that PCPs serve as default mental health providers despite insufficient training [16]. Of note, there were regional differences in this study, with more providers in Southern Maryland reporting discomfort prescribing psychiatric medications compared to the other regions. This may be due to the over-representation of nurse respondents in that region, who are not able to prescribe psychiatric medications. Additionally, nearly all respondents in Southern Maryland and 80% in Western Maryland reported difficulty finding CAPs, which is consistent with workforce data indicating significant shortages (i.e., ~3 CAPs/10,000 children) in these regions [12].

Like many child psychiatry access programs, BHIPP has taken steps to address gaps in pediatric mental health services by providing standalone 1- to 3-hour trainings, consultation, and resource/referral services to Maryland PCPs. Results of this needs assessment align with recent BHIPP warmline utilization; referral calls reflect a higher proportion of calls to BHIPP in each of the last 2 years compared to calls for consultation, and medication evaluation/change has been the most common reason for consultation. Results underscore the importance of expanding the mental health workforce and access to specialty mental health care. In particular, results highlight the need for additional longitudinal training and consultation from CAPs and psychologists to expand PCPs’ mental health treatment capacity, as well as the urgent need for increased access to direct mental health treatment provided by specialists (e.g., CAPs, psychologists) through use of promising integrated care models, such as in-person evaluation/treatment with mental health providers co-located with PCPs or at an agency partnering with the primary care site, and tele-mental health services (e.g., telepsychiatry) [5]. Of note, data from this study were collected prior to March 2020 and do not reflect mental health service access during the COVID-19 pandemic. COVID-19 is expected to increase the need for mental health care, underscoring the importance of child psychiatry access program services to support pediatric PCPs and their patients [17].

There were some limitations to this study. Although 107 providers participated, respondents represented just under half of practices in target regions, with variation in the proportion of participating providers per practice leading to underrepresentation from some practices and overrepresentation from others. Thus, findings may not be representative of all PCPs or practices in these regions and should be interpreted cautiously due to possible selection bias [18]. Although the total number and demographic characteristics of all providers practicing at each practice in these regions is currently unknown, BHIPP continues to identify PCPs within the target regions, and has been able to reach a substantial proportion of the pediatric primary care workforce. However, the lack of information about non-responders limits our ability to evaluate the extent of selection bias in this study. Additionally, results may not generalize to other regions and states as the target regions reflect three high-need, rural regions with very limited access to mental health providers, particularly CAPs [12]. Unique geographic differences in these regions affect access to mental health services, with the Appalachian Mountains passing through one and the Chesapeake Bay separating the others. It is notable, however, that the sample, which is majority White and female, is representative of the larger pediatric primary care workforce and those contacting child psychiatry access programs [11, 19].

Guided by needs assessment results, BHIPP aims to increase availability and accessibility of pediatric behavioral health treatment through the provision of tele-mental health services, care coordination, and web-based longitudinal training using the Project ECHO® (Extension for Community Healthcare Outcomes) model [20]. Tele-mental health services will include consultation and direct service delivery provided to patients within primary care sites across Maryland via real-time videoconferencing [5]. These services will be provided by CAPs and social workers based on patient needs, allowing for a broad range of mental health services over real-time videoconferencing. Care coordination will also be available to providers and families for resource/referral assistance and follow-up over the phone. Project ECHO is a widely used telementoring model that aims to address the inadequate supply of specialty care in rural and underserved areas through case-based learning and didactic education via videoconferencing to front-line health care workers in those regions to increase their capacity to provide best-practice care [20]. BHIPP’s ECHO didactic curriculum and case-specific guidance will be directly informed by the mental health practices identified as areas of discomfort (e.g., prescribing psychotropic medication) by respondents in this study. Through these expanded services, BHIPP aims to increase PCP knowledge and comfort with a broader range of mental health practices as well as augment access to specialty mental health care through tele-mental health services. Future work will evaluate the impacts of these expanded services on provider and patient outcomes to guide best practices for increasing access to pediatric mental health services.
